# Advances in Medical Education: The Importance of Communication and Collaboration

**Published:** 2012-03-31

**Authors:** Tanya Beran

**Affiliations:** University of Calgary, Calgary, Canada

At the core of many discussions about advances in medical education is communication/collaboration. This ability is also the foundation of clinical competence. According to Epstein and Hundert,^1^ competence in medicine is, “the habitual and judicious use of communication, knowledge, technical skills, clinical reasoning, emotions, values, and reflection in daily practice for the benefit of the individuals and communities being served.” The practice of medicine takes place in a social context, requiring the physician to have strong interpersonal skills to interact effectively with patients, their families, and other healthcare professionals. But this is not a new idea. In this issue of CMEJ we situate communication/collaboration both within and outside of the curriculum. In some studies, the reader will see that this topic fits squarely within the curriculum, whereas others show communication improvement activities to be external to standard training. Yet, others are an extension of the curriculum. Most of the studies published here empirically demonstrate the importance of the physician’s interaction within inter-professional groups.

We begin our articles with Price et al., who report survey responses from nurses and anesthesiologists that emphasize the need for team training in realistic situations such as code blue emergencies. They further specify the need to communicate the details of the situation succinctly and quickly, with clear identification of leadership roles. In their conclusions, they describe the “SBAR” organizational framework as a model of communication for emergency situations, which exemplifies the type of interaction identified in their needs analysis. Thus, this paper not only points out the importance of communication/collaboration, according to nurses’ and anesthesiologists’ perspectives, but it also provides a solution of how to respond to this identified need. In the subsequent paper, Wong addressed the call to create a functional and adequate postgraduate education program in ambulatory care for internal medicine. Through the Delphi method, agreement was reached on the core competencies for each of the CanMEDS roles, such as communicator and collaborator. As provided in several tables, these competencies can serve to guide development of curriculum development and assessment.

Kornelsen and colleagues lead our presentation of communication towards the context of mentorship. In their qualitative analysis of interviews with general practitioner surgeons in rural areas, these researchers found that mentorship was the most influential reason for choosing a rural practice. It is particularly relevant when there is no curriculum built into their medical training pertaining to rural medicine. Mentorship, then, may be the sole opportunity for students to consider rural practice.

Alansari further explicates the importance of effective communication in his introduction – namely for high quality patient care. He then reported differences in communication skills between general surgery residents and general practitioner residents, with the former performing higher on history and diagnosis but the latter performing higher on information-sharing with the patient. Although the overall level of communication skills was high, Alansari concluded that increased attention be devoted in medical education to effective communication with patients.

Communication and collaboration are also applicable to the study conducted by Loewen and colleagues in their survey of radiation oncologists to assess employability in Canada. Although hiring in the Canadian workforce is currently strong, these authors expressed concern about the need for valid projections of the needed workforce in years to come that is consistent with the rate of graduation. This type of estimation and coordination is needed in most, if not all, healthcare sectors and suggests that communication and collaboration are important for career planning, in addition to patient care. In the subsequent paper, François points out the importance not only for strong verbal, but also written, communication ability. A self-study package with guidelines on effective writing was given to family medicine residents. The author appropriately suggests that the resulting improvement in writing skills may indeed reflect the residents’ gains in understanding consultation and referral – the primary purpose of communication through writing. As with the Komelsen et al. study, Turner and his colleagues concur that access to role models would influence students’ selection of specialization. Specifically, directors of pre-clinical undergraduate medical education were generally in agreement that surgeons should be included more in the teaching of students. The authors suggest that this involvement may increase student interest in surgery, which research suggests is in decline.^2^

Curriculum transformation is the topic for the next article. Hasan and Sequeira provide their perspective and detailed account on how to re-organize a curriculum in physiology. They address many guiding goals of this process, such as setting clear objectives for the curriculum, integrating information and disciplines, and ongoing assessment. One of their conclusions is that collaborative input is necessary for such a major endeavour.

The challenges for International Medical Graduates are discussed in two articles. First, Obara comments on the residency application for international medical graduates. His response to *The Canadian International Medical Graduate Bottleneck: A New Problem for New Doctors* elucidates the reason why Canadian international medical graduates (Canadian citizens who study medicine abroad) are less likely to receive a residency position in Canada than non-Canadian graduates. The eligibility of the applications were explained as a ‘timing’ issue, that did not allow the Canadian international graduates opportunity to apply for a residency position. Second, Duncan and Poddar continue this discussion by comparing international medical graduates to Canadian-trained physicians. They point out that the former tend to have fewer opportunities to gain experience in the Canadian clinical setting, leaving them feeling ill-prepared for residency. Thus, it would seem that greater coordination of the licensure process for all international medical graduates is sorely needed.

This issue is concluded with Hebbal et al.’s question about the leadership qualities of dentists. Essentially, they argue it is *expected* that leadership programs be embedded within the curriculum, given the need to adapt to economic, political, and technological changes in society. As can be seen in this issue, a stronger emphasis on communication and improved coordination of medical education programs and curricula are essential for physician preparation.
